# Ultrasound-Responsive Drug Delivery System Based on Piezoelectric Catalytic Mechanisms

**DOI:** 10.3390/jfb16080304

**Published:** 2025-08-21

**Authors:** Kaixi Cui, Tianzheng Li, Yifei Ma, Chuanjin Zhang, Ke Zhang, Chao Qi, Kaiyong Cai

**Affiliations:** 1 Hongshen Honors School, Chongqing University, Chongqing 400044, China; 20226175@stu.cqu.edu.cn; 2 Key Laboratory of Biorheological Science and Technology, Ministry of Education College of Bioengineering, Chongqing University, Chongqing 400044, China; 20225196@stu.cqu.edu.cn (T.L.); 20233641@stu.cqu.edu.cn (C.Z.); 3 School of Electrical Engineering, Chongqing University, Chongqing 400044, China; 20220949@stu.cqu.edu.cn

**Keywords:** ultrasound response, drug delivery, piezoelectric catalysis, ROS-sensitive hydrogel

## Abstract

Ultrasound-responsive nanomaterials represent a promising approach for achieving non-invasive and localized drug delivery within tumor microenvironments. In this study, we developed a piezocatalysis-assisted hydrogel system that integrates reactive oxygen species (ROS) generation with stimulus-responsive drug release. The platform combines piezoelectric barium titanate (BTO) nanoparticles with a ROS-sensitive hydrogel matrix, forming an ultrasound-activated dual-function therapeutic system. Upon ultrasound irradiation, the BTO nanoparticles generate ROS—predominantly hydroxyl radicals (^•^OH) and singlet oxygen (^1^O_2_)—through the piezoelectric effect, which triggers hydrogel degradation and facilitates the controlled release of encapsulated therapeutic agents. The composition and kinetics of ROS generation were evaluated using radical scavenging assays and fluorescence probe techniques, while the drug release behavior was validated under simulated oxidative environments and acoustic fields. Structural and compositional characterizations (TEM, XRD, and XPS) confirmed the quality and stability of the nanoparticles, and cytocompatibility was assessed using 3T3 fibroblasts. This synergistic strategy, combining piezocatalytic ROS generation with hydrogel disintegration, demonstrates a feasible approach for designing responsive nanoplatforms in ultrasound-mediated drug delivery systems.

## 1. Introduction

Malignant tumors continue to represent one of the most significant global health challenges due to their high mortality and recurrence rates. Although conventional treatments, such as surgery, radiotherapy, and chemotherapy, are fundamental in clinical oncology, they are frequently constrained by systemic toxicity, drug resistance, and limited tumor selectivity [1,2,3]. To address these limitations, extensive research has been directed toward the development of stimulus-responsive nanomaterials [4,5] aimed at enhancing the precision and effectiveness of drug delivery in cancer therapy [6,7,8].

Among various external stimuli, ultrasound has garnered considerable attention due to its advantages, including deep tissue penetration, non-invasiveness, and spatiotemporal controllability. Ultrasound-responsive drug delivery systems allow for controlled drug release and have demonstrated potential in improving tumor selectivity [9].

One promising strategy involves the application of piezoelectric nanomaterials, such as barium titanate (BaTiO_3_), which produce electrical charges in response to mechanical or acoustic stimulation. The resulting piezoelectric effect can induce redox reactions at the nanoparticle surface, leading to the generation of reactive oxygen species (ROS), including hydroxyl radicals (^•^OH) and singlet oxygen (^1^O_2_). These ROS can trigger oxidative stress in cancer cells and act as therapeutic mediators in sonodynamic therapy (SDT) [10,11,12].

In parallel, ROS-responsive hydrogels have emerged as intelligent drug delivery systems that degrade in oxidative environments. These hydrogels are typically synthesized using excipients such as phenylboronic acid derivatives, which form dynamic boronate ester linkages with polyol-containing polymers [13]. One representative compound is trimethylphenylboronic acid (TPBA), which contains multiple boronic acid groups and facilitates crosslinking via reversible covalent bonds. These bonds are selectively cleaved in the presence of ROS, particularly ^•^OH, resulting in hydrogel structural disintegration and the subsequent release of encapsulated therapeutic agents [13].

Despite significant advances in SDT and responsive hydrogel systems, the integration of piezoelectric-induced ROS generation [14] with ROS-sensitive hydrogel degradation to enable controlled drug release remains largely unexplored. While several studies have reported acoustic-activated ROS systems, key challenges persist in achieving synchronized activation, efficient ROS generation, and on-demand drug delivery.

**Figure 1:**
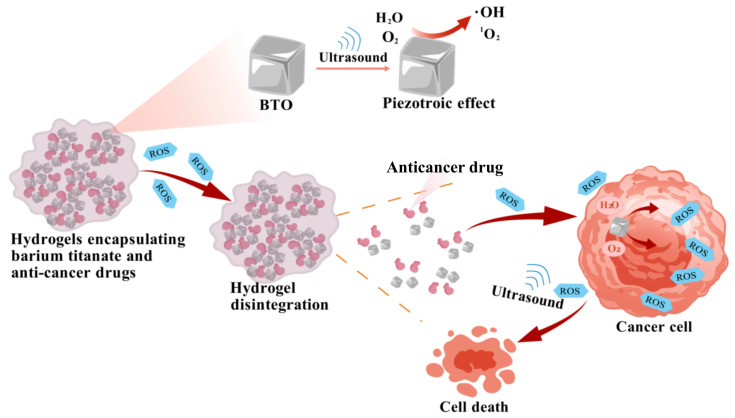
Schematic diagram of ultrasound-responsive controlled release drug system based on piezoelectric catalysis.

In this study, we aim to develop a composite system that integrates BaTiO_3_ nanoparticles with a ROS-sensitive hydrogel network to achieve ultrasound-triggered, site-specific drug release. The hydrogel is designed to degrade under conditions of elevated ROS—either endogenous (tumor-derived) or exogenous (ultrasound-induced). Rhodamine B is employed as a model compound to assess drug release behavior [15]. All experiments are conducted in vitro to simulate the oxidative stress of the tumor microenvironment and to evaluate the feasibility, responsiveness, and therapeutic potential of the system. (Figure 1)

## 2. Materials and Methods

### 2.1. Experimental Materials

#### 2.1.1. Materials

Titanium tetrabutoxide (Ti[O(CH_2_)_3_CH_3_], Sigma-Aldrich, St. Louis, MO, USA), ethanol (analytical grade, Chongqing Chuandong Chemical (Group) Co., Ltd., Chongqing, China), 25% aqueous ammonia (NH_4_OH solution, Shanghai Aladdin Biochemical Technology Co., Ltd., Shanghai, China), barium hydroxide monohydrate (Ba(OH)_2_·H_2_O, Shanghai Macklin Biochemical Co., Ltd., Shanghai, China), acetic acid (Shanghai Macklin Biochemical Co., Ltd., Shanghai, China), tert-butanol (TBA, Shanghai Aladdin Biochemical Technology Co., Ltd., Shanghai, China), furfuryl alcohol (FFA, Shanghai Macklin Biochemical Co., Ltd., Shanghai, China), p-benzoquinone (pBQ, Shanghai Macklin Biochemical Co., Ltd., Shanghai, China), singlet oxygen sensor green (SOSG, Shanghai Biyuntian Biological Co., Ltd., Shanghai, China), nitroblue tetrazolium chloride (NBT, Shanghai Macklin Biochemical Co., Ltd., Shanghai, China), terephthalic acid (TA, Shanghai Aladdin Biochemical Technology Co., Ltd., Shanghai, China), N,N,N′,N′-tetramethyl-1,3-propanediamine (Shanghai Aladdin Biochemical Technology Co., Ltd., Shanghai, China), 4-(bromomethyl)phenylboronic acid (Shanghai Aladdin Biochemical Technology Co., Ltd., Shanghai, China), tetrahydrofuran (THF, Shanghai Aladdin Biochemical Technology Co., Ltd., Shanghai, China), polyvinyl alcohol (PVA, Shanghai Aladdin Biochemical Technology Co., Ltd., Shanghai, China), phosphate-buffered saline (PBS buffer, Biosharp, Hefei, China), 10 mM hydrogen peroxide (H_2_O_2_, Shanghai Aladdin Biochemical Technology Co., Ltd., Shanghai, China) solution, rhodamine B isothiocyanate (RBITC) fluorescent reagent (Shanghai Aladdin Biochemical Technology Co., Ltd., Shanghai, China), DMEM complete culture medium (Procell Life Science & Technology Co., Ltd., Wuhan, China), serum-free culture medium, and Cell Counting Kit-8 reagent (UElandy, Suzhou, China).

#### 2.1.2. Characterization of BTO

Barium titanate nanoparticles were synthesized as described and characterized for their physicochemical properties [16]. Prior to observation, the nanoparticles were dispersed in absolute ethanol and sonicated for 15 min. A 5 μL aliquot of the suspension was then dropped onto a carbon-coated copper TEM grid (300 mesh) and allowed to air-dry at room temperature. Transmission electron microscopy (TEM) and high-resolution TEM (HRTEM) were performed using a Talos™ F200S field emission instrument (FEI, Hillsboro, USA), operated at an accelerating voltage of 200 kV. Bright-field and lattice-resolved images were acquired using a Gatan Ceta CMOS camera (Thermo Fisher Scientific, Waltham, MA, USA), and selected area electron diffraction (SAED) patterns were obtained to assess crystallinity. The instrument was equipped with an Oxford X-MaxN 60 mm^2^ energy-dispersive X-ray spectroscopy (EDS, Thermo Fisher Scientific, Waltham, MA, USA) detector for elemental mapping. X-ray diffraction (XRD, Thermo Fisher Scientific, Waltham, MA, USA) patterns were recorded using a PANalytical X’ Pert PRO diffractometer (Netherlands). X-ray photoelectron spectroscopy (XPS) was conducted on an ESCALAB 250Xi system (Thermo Fisher Scientific, Waltham, MA, USA) to analyze surface elemental composition and chemical states. The hydrodynamic diameter and zeta potential of the nanoparticles were measured using a Zetasizer Nano ZS90 (Malvern Instruments Ltd., Worcestershire, UK). Ultraviolet-visible (UV-Vis) absorption spectra were obtained with a NanoDrop^TM^ One spectrophotometer (Thermo Fisher Scientific, Waltham, MA, USA). Ultrasound-induced temperature variation was monitored using a digital thermometer (Testo 925, Lenzkirch, Germany) under identical acoustic exposure conditions used in experimental treatments [17,18,19,20].

### 2.2. Synthesis of BTO Nanoparticles

In this experiment, barium titanate nanoparticles (BTO NPs) were prepared using a typical hydrothermal reaction. First, 17.018 g (50 mM) of titanium tetrabutoxide was mixed with 20 mL of ethanol (analytical grade purity) and 7 mL of ammonium hydroxide solution (25% ammonia water). Then, 14.204 g (75 mM) of Ba(OH)_2_·H_2_O dissolved in 25 mL deionized water (boiling water bath) was added. The two suspensions were mixed (heated mixing) and transferred into a stainless steel autoclave lined with 100 mL polytetrafluoroethylene and then heat-treated at 200 °C for 48 h. After the reaction, the resulting product was repeatedly washed with 5% acetic acid solution and anhydrous ethanol and then dried in an oven at 80 °C for 24 h to obtain the desired piezoelectric material barium titanate nanoparticles. A portion of the synthesized barium titanate piezoelectric material was taken for characterization testing [16].

### 2.3. COMSOL Physical Field Simulation

COMSOL(6.2) physical field simulation aims to employ finite element physical field simulation technology for in-depth analysis of the electric potential of piezoelectric material in sonodynamic therapy [21,22,23]. Specifically, we will simulate the piezoelectric response of cubic barium titanate (BTO) nanocrystals with edge lengths of 100 nm immersed in an aqueous medium (simulating the intracorporeal environment), under ultrasound exposure with an intensity of 4 × 10^9^ Pa and a frequency of 1 MHz. This simulation is intended to verify whether barium titanate can generate a piezoelectric potential difference exceeding 0.25 V (the minimum threshold potential required for generating reactive oxygen species) in an aqueous environment.

### 2.4. Investigation of the Piezocatalysis Performance and Mechanism of BTO

#### 2.4.1. Radical Scavenging Experiment

The experiment was divided into the following four groups: “control group”, “tert-butanol (TBA) group”, “furfuryl alcohol (FFA) group”, and “p-benzoquinone (pBQ) group” [6,8]. First, 8 mL of methylene blue (MB) (5 mg/L) was added, respectively, to each of the four groups as the reaction substrate. Each group then received 8 mg of BTO NPs. Subsequently, 0.59 mg of tert-butanol, 0.78 mg of furfuryl alcohol, and 0.86 mg of p-benzoquinone were, respectively, added to the three groups, excluding the control group, followed by pipetting to ensure complete mixing. The four mixtures were subjected to ultrasound treatment, and samples were collected at 0 min, 2 min, 4 min, 6 min, 8 min, and 10 min. Each sample was centrifuged for 10 min at a speed of 10,000 r/min to prevent interference from BTO NPs during fluorescence detection. The supernatant of each centrifuged reagent was collected for absorbance measurement.

#### 2.4.2. Radical Specificity Test

The experiment was divided into the following three groups: a “TA group”, an “SOSG group”, and an “NBT group” [24]. First, 35 mL of BTO NP solution with a concentration of 0.2 mol/mL was prepared using distilled water. Each group received 8 mL of the BTO NP solution. Then, 80 μL of TA solution (10 mg/mL, dissolved in NaOH), 80 μL of SOSG solution (10 mg/mL, dissolved in water), and 80 μL of NBT solution (10 mg/mL, dissolved in water) were, respectively, added to the three groups, followed by shaking and mixing thoroughly. The three mixed solutions were subjected to ultrasound treatment (1 MHz, 1 W/cm^2^), and samples were collected at 0 min, 2 min, 4 min, 6 min, 8 min, and 10 min. The collected samples were centrifuged for 10 min at a speed of 10,000 r/min using a centrifuge to avoid interference from BTO NPs on fluorescence detection. The supernatant of the centrifuged reagents was collected and analyzed for fluorescence intensity using a fluorescence spectrophotometer.

### 2.5. Synthesis of ROS-Responsive Hydrogel

Firstly, trimesic phenylboronic acid was synthesized: N,N,N′,N′-tetramethyl-1,3-propanediamine (0.1 g, 0.75 mM) and 4-(bromomethyl)phenylboronic acid (0.5 g, 2.3 mM) were dissolved in DMF (10 mL). After stirring at 60 °C for 24 h, the mixture was poured into 100 mL of tetrahydrofuran (THF), resulting in a white precipitate. The mixture was centrifuged at 9000 rpm for 8 min to remove the supernatant. The precipitate was then washed with THF three times following the same procedure. Finally, the product was dried under vacuum overnight to obtain purified trimesic phenylboronic acid. The hydrogel composite was prepared by mixing a solution containing the fluorescent model drug (rhodamine B, 1 mg/mL), barium titanate nanoparticles (10 mg/mL), and polyvinyl alcohol (10 wt%) with the synthesized small molecule solution of trimesic phenylboronic acid (50 mg/mL) at a 1:1 volume ratio to form a gel.

### 2.6. Release Experiment of Hydrogel Composite

#### 2.6.1. Drug Release of Composite Hydrogel Under ROS-Responsive Conditions

To investigate the effect of different ROS concentrations on the disintegration and drug release of hydrogels, this experiment was divided into a “PBS group (simulating normal human body fluid environment, low ROS concentration)” and “H_2_O_2_ group (simulating tumor microenvironment, high ROS concentration)” for release detection.

Equal amounts of hydrogels encapsulating the hydrophilic fluorescent reagent rhodamine (as a substitute for the anticancer drug) were separately placed into PBS solution (simulating a normal human body fluid environment) and 10 mM H_2_O_2_ solution (simulating a high ROS concentration environment). All samples were placed on a shaker at 37°, and aliquots were collected at 0 h, 0.5 h, 1 h, 2 h, 4 h, 8 h, 12 h, 24 h, 48 h, and 72 h. Fluorescence intensity of each sample was measured using a fluorescence spectrophotometer.

#### 2.6.2. Drug Release of Composite Hydrogel Under Ultrasound Conditions

In this experiment, one group was set as pure hydrogel, and another group was set as hydrogel composite with added BTO NPs. Pure hydrogel (Gel) group: 500 μL of trimesic phenylboronic acid small molecule solution was thoroughly mixed with 500 μL of PVA solution. BTO NPs + hydrogel (BTO@Gel) group: 2 mg of BTO NPs was weighed and mixed with 2 mL of trimesic phenylboronic acid small molecule solution. A total of 500 μL of the above-mentioned mixture was taken and thoroughly mixed with 500 μL of PVA solution. To facilitate subsequent data collection, equal amounts of fluorescent reagent were encapsulated in both groups. Both groups were then subjected to ultrasound treatment at 1 W/cm^2^, 1 MHz for 10 min, followed by oscillation for 30 min. After standing for 1.5 h, ultrasound treatment was repeated for another 10 min. Subsequently, both groups were placed into dialysis bags containing 20 mL of phosphate-buffered saline (PBS) for drug release testing (samples were collected at 0 h, 0.5 h, 1 h, 2 h, 4 h, 24 h, 48 h, and 72 h). Fluorescence intensity of each sample collected at different time points was then measured using a fluorescence spectrophotometer.

### 2.7. Cell Safety Experiment

This study selected the 3T3 mouse embryonic fibroblast cell line (Pricella Life Science & Technology Co., Ltd., Wuhan, China) as the biological safety evaluation model. The 3T3 cells were seeded at a density of 1 × 10^4^ cells/well in 48-well cell culture plates, with each well containing 200 μL of complete DMEM medium supplemented with 10% fetal bovine serum. Cells were pre-cultured for 24 h at 37 °C and 5% CO_2_ to achieve a cell adhesion rate of 80% ± 5%. Experimental groups received 20 μL of gradient concentrations (25, 50, 100, 200, 300, 400, and 500 μg/mL) of barium titanate nanoparticles (BTO) or 20 μL of leaching solutions containing gradient concentrations (25, 50, 100, 200, 300, 400, and 500 μg/mL) of hydrogel-encapsulated BTO materials, where the leaching solution was subjected to ultrasound treatment (1 W/cm^2^, 1 MHz) for 10 min. The control group received an equal volume (20 μL) of PBS. After co-culturing 3T3 cells with gradient concentrations of BTO materials and hydrogel-encapsulated gradient concentrations of BTO leaching solution for 24 h, the cell viability was analyzed using a modified Cell Counting Kit-8 protocol: remove the culture medium carefully without detaching the monolayer cells, rinse twice gently with pre-warmed PBS (pH 7.4), and thoroughly remove residual medium to prevent interference. Then, add a mixture of serum-free medium and Cell Counting Kit-8 reagent at a 9:1 volume ratio and incubate in the dark at 37 °C for 30 min. Following incubation, transfer the liquid to a 96-well plate and measure the optical density (OD) using a microplate reader (BioTek Synergy H1) at a primary wavelength of 450 nm.

### 2.8. Statistical Analysis

All quantitative data were expressed as mean ± standard deviation (SD). Cell viability assays, zeta potential, particle size, and thermal effect assessments were conducted in triplicate (*n* = 3). Statistical analysis was performed using one-way ANOVA followed by Tukey’s post hoc test. A *p*-value < 0.05 was considered statistically significant. Before applying ANOVA, data were tested for normality using the Shapiro–Wilk test and for homogeneity of variances using the Brown–Forsythe and Bartlett’s tests. These analyses were conducted using GraphPad Prism 9.0 software (GraphPad Software, San Diego, USA). Other experiments involving ROS detection and drug release were performed as proof-of-concept studies conducted once, unless otherwise specified.

## 3. Results and Discussion

### 3.1. Synthesis and Characterization of BTO Nanoparticles

To comprehensively assess the physicochemical properties of the synthesized barium titanate (BaTiO_3_ and BTO) nanoparticles prepared as described in Section 2.1.2, a series of multi-scale characterization techniques was employed, including structural, morphological, and surface chemical analyses [9,10,14]. The results confirmed that the synthesized BTO nanoparticles met the expected structural and compositional standards.

**Figure 2:**
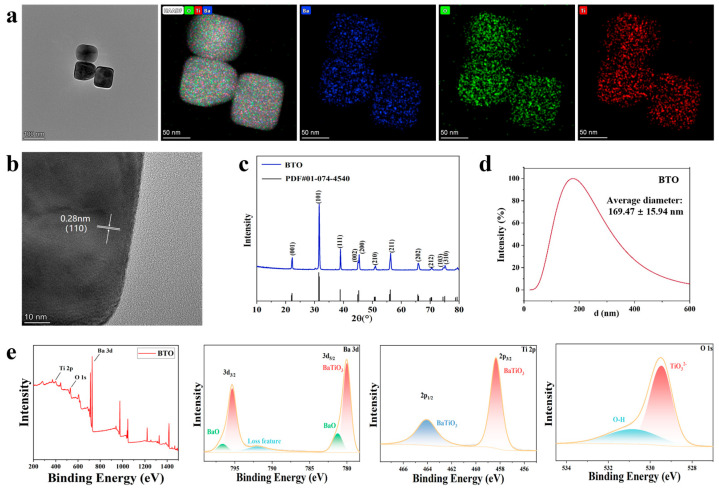
Morphological and structural characterizations of BTO. (**a**) TEM image, (**b**) HRTEM image, (**c**) X-ray powder diffraction image, (**d**) particle size distribution (*n* = 3), (**e**) X-ray photoelectron spectroscopy image and its fitting peak.

Transmission electron microscopy (TEM) revealed that the BTO nanoparticles exhibited a uniform cubic morphology with a narrow size distribution of approximately 50 ± 5 nm (Figure 2a). High-resolution TEM (HRTEM) further confirmed the crystalline nature of the particles, displaying a clear lattice fringe with an interplanar spacing of 0.28 nm corresponding to the (110) crystal plane (Figure 2b).

X-ray diffraction (XRD) analysis confirmed the tetragonal phase structure of BTO. The diffraction peaks observed at 2θ = 31.5°, 38.8°, and 45.3° (Figure 2c) matched well with the standard pattern of tetragonal BTO (space group P4mm), with no detectable impurity peaks, indicating high crystallinity and phase purity.

X-ray photoelectron spectroscopy (XPS, Figure 2e) was conducted to analyze the surface chemical states. The Ba 3d spectrum displayed characteristic peaks at 779.2 eV (Ba 3d_5/2_) and 794.5 eV (Ba 3d_3/2_), confirming the presence of Ba^2+^. The Ti 2p region showed doublet peaks at 458.7 eV (Ti 2p_3/2_) and 464.4 eV (Ti 2p_1/2_), corresponding to Ti^4+^. The O 1s peak at 529.6 eV was assigned to lattice oxygen (O^2−^), further supporting the stoichiometry of BTO. Elemental analysis of the XPS data showed atomic compositions of O (69.01%) > Ti (17.55%) > Ba (13.44%), closely matching the theoretical stoichiometric ratio of BaTiO_3_. Additionally, the symmetric peak shapes and narrow full width at half maximum (FWHM) indicated uniform chemical environments and the absence of surface contamination or secondary phases.

Moreover, BTO nanoparticles exhibited an average hydrodynamic diameter of 169.47 ± 15.94 nm (Figure 2d), while the zeta potential measured in PBS at physiological pH 7.4 was −13.11 ± 2.51 mV (Table 1).

Through various characterization methods, such as TEM, HRTEM, XRD, and XPS, we confirmed that the prepared BTO nanoparticles possess a tetragonal crystal structure, and their microstructure, crystal phase, and surface chemical states are consistent with expectations.

### 3.2. COMSOL Physical Field Simulation Results

**Figure 3:**
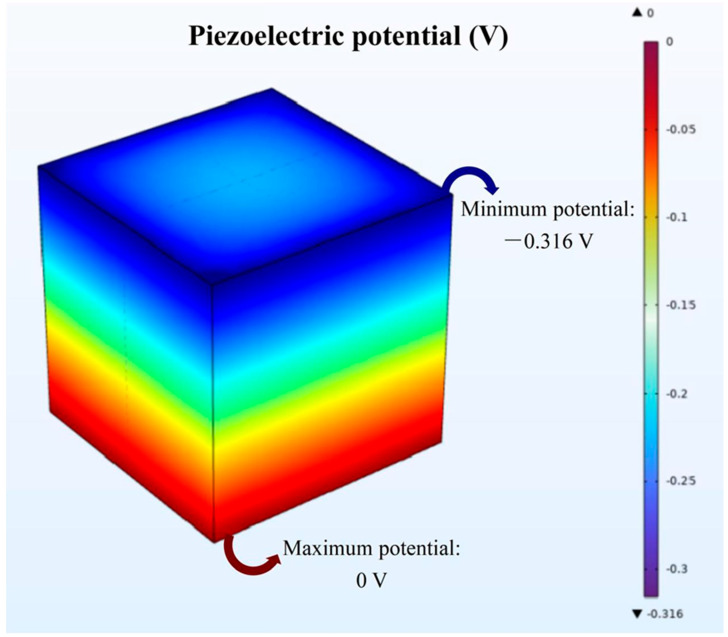
COMSOL physical field simulation results.

To theoretically validate the piezocatalytic potential of BTO nanoparticles under ultrasonic excitation, an acoustoelectric coupled model was constructed using the COMSOL Multiphysics platform. This simulation aimed to analyze the spatial distribution of the piezoelectric potential generated within an ultrasonic field, thereby providing theoretical support for subsequent experimental investigations. The simulation parameters were set as follows: an acoustic pressure of 4 × 10^9^ Pa and a directional ultrasound excitation frequency of 1 MHz. As illustrated in Figure 3, the model predicted a significant piezoelectric potential difference across the surface of BTO nanoparticles, with a maximum value reaching 0.316 V. This exceeds the critical threshold potential (~0.25 V) required to initiate piezocatalytic redox reactions. The generated potential was sufficient to induce band bending, promote the separation and migration of electron–hole pairs, and drive surface redox reactions. These processes collectively facilitate the formation of ROS, including ^•^OH and ^1^O_2_, under acoustic stimulation.

### 3.3. BTO NP Performance and Mechanism Investigation

#### 3.3.1. Identification of the Dominant ROS via Radical Scavenging Assays

To identify the predominant types of ROS generated by BTO nanoparticles under ultrasonic stimulation, radical scavenging experiments were performed using methylene blue (MB) as a chromogenic probe. The degradation of MB was employed as an indirect indicator of ROS activity within the system.

The experiment was divided into four groups based on the specific reagents added to target different types of ROS: a control group (no radical-specific reagent added), tert-butanol (TBA, scavenger for ^•^OH), furfuryl alcohol (FFA, scavenger for ^1^O_2_), and p-benzoquinone (pBQ, scavenger for superoxide anion O_2_^−^).

Upon ultrasonic excitation, BTO nanoparticles continuously generated ROS, resulting in MB degradation, as indicated by a decrease in absorbance. When a specific scavenger was added, it selectively neutralized the corresponding radical species, thereby reducing the extent of MB degradation. Consequently, a smaller absorbance change reflected a greater contribution of the respective radical to the overall ROS activity.

**Figure 4:**
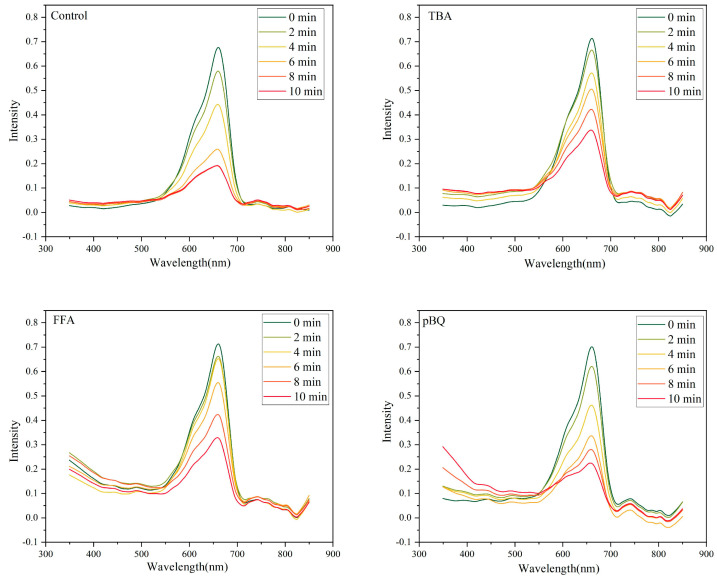

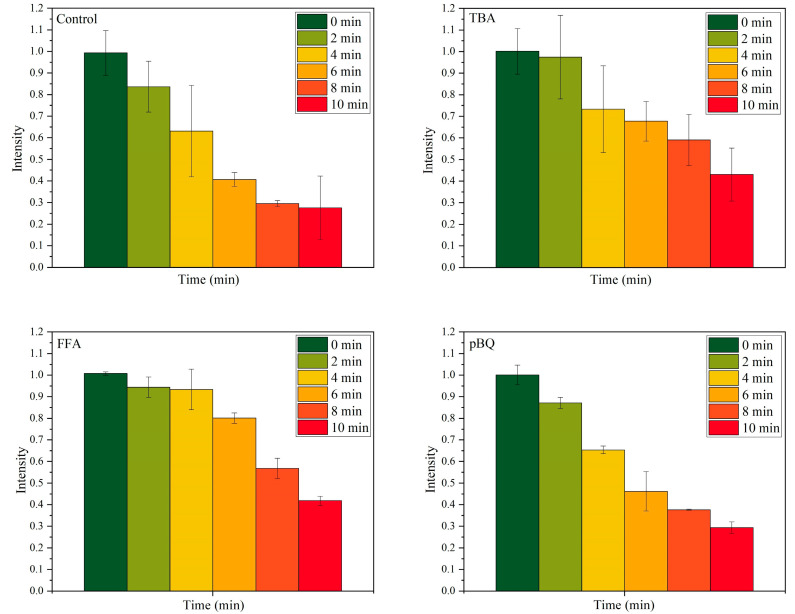
Free radical quenching experiment to explore the main ROS species. Data are presented as mean ± SD (*n* = 3).

As shown in Figure 4, the absorbance of MB in TBA and FFA scavenger-treated groups decreased over time, indicating the involvement of ^•^OH and ^1^O_2_ in ROS-mediated degradation. Notably, the TBA group exhibited the slowest degradation rate and the least reduction in absorbance, suggesting that ^•^OH played a dominant role. In contrast, the degradation of MB in the pBQ group was comparable to that in the control group, suggesting that the O_2_^−^ contributed minimally to the overall ROS activity.

#### 3.3.2. Validation of the Dominant ROS via Specific Fluorescent Probes

Following the identification of the dominant ROS through radical scavenging experiments, a complementary verification was performed using radical-specific fluorescent probes to quantitatively assess the generation of individual ROS types. This method relies on the principle that different ROS species react with specific probes to produce highly fluorescent products, thereby enabling indirect quantification of ROS levels based on fluorescence intensity.

**Figure 5:**
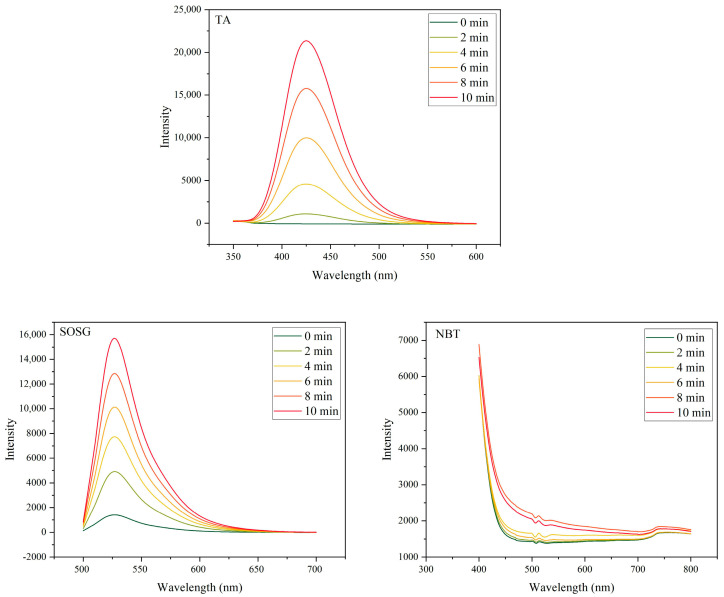
Free radical specificity test to explore the main ROS species.

Under the sonication conditions defined in Section 2.4.2 (fixed frequency and time intervals), fluorescence intensities of the supernatants were measured across groups using three probe systems: terephthalic acid (TA) for ^•^OH, singlet oxygen sensor green (SOSG) for ^1^O_2_, and nitroblue tetrazolium (NBT) for O_2_^−^, as shown in Figure 5.

The results demonstrated a significant increase in fluorescence intensity in the TA and SOSG groups with prolonged sonication time, indicating substantial generation of ^•^OH and ^1^O_2_ in the reaction system. In contrast, the NBT group exhibited a relatively weaker fluorescence response, suggesting limited formation of O_2_^−^. These findings are consistent with the radical scavenging results, further confirming that ^•^OH and ^1^O_2_ are the predominant ROS generated by BTO under ultrasound excitation.

### 3.4. Verification and Analysis of the Release Performance of Composite Hydrogels

As described in Section 2.5, a ROS-responsive hydrogel composite was synthesized by incorporating BTO nanoparticles and rhodamine B, a hydrophilic fluorescent dye used as a model drug. To verify the functional mechanism of the system, two sequential experiments were conducted: first, the effect of ROS concentration on hydrogel degradation was evaluated; second, it was investigated whether BTO nanoparticles could promote drug release under ultrasound stimulation through ROS generation. This experimental design was informed by COMSOL simulation results, which confirmed that BTO can generate ROS under a specific acoustic field. Therefore, enhanced hydrogel degradation and rhodamine B release under ultrasound would serve as experimental evidence supporting the proposed mechanism—namely, that BTO-induced ROS production triggers hydrogel disintegration and facilitates controlled drug release.

#### 3.4.1. Drug Release Behavior of the Composite Hydrogel Under H_2_O_2_ Conditions

**Figure 6:**
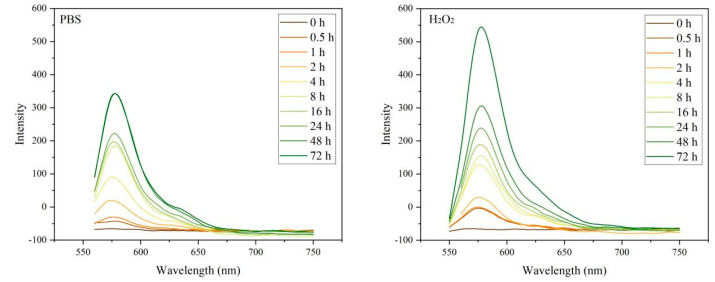
Under the environment of PBS and H_2_O_2_ (10 mmol/L), the fluorescence intensity at different times.

To assess the ROS responsiveness of the hydrogel system developed in this study, an in vitro simulation of the tumor microenvironment was performed by introducing elevated ROS levels. The hydrogel’s degradation behavior and drug release performance were evaluated under different oxidative stress conditions. Since the anticancer drug used in the formulation lacks intrinsic fluorescence, rhodamine B—a hydrophilic fluorescent molecule with similar physicochemical properties and diffusion behavior—was employed as a model compound to simulate drug release. Changes in fluorescence intensity served as indirect indicators of hydrogel disintegration and release efficiency [25]. Under consistent incubation times, supernatants from each experimental group were collected and analyzed for fluorescence intensity. As shown in Figure 6, the phosphate-buffered saline (PBS) group exhibited a peak fluorescence intensity of approximately 350. In contrast, the group treated with hydrogen peroxide (H_2_O_2_) showed a markedly enhanced fluorescence signal, peaking at around 550. This result indicates that high ROS levels significantly accelerated hydrogel breakdown and promoted the release of encapsulated agents [26].

In conclusion, the results demonstrate that the hydrogel system possesses excellent ROS responsiveness, enabling accelerated structural disintegration and payload release under elevated oxidative stress. This behavior provides a robust foundation for achieving precise and controllable drug delivery within the tumor microenvironment.

#### 3.4.2. Drug Release Properties of Composite Hydrogels Under Ultrasound Stimulation

**Figure 7:**
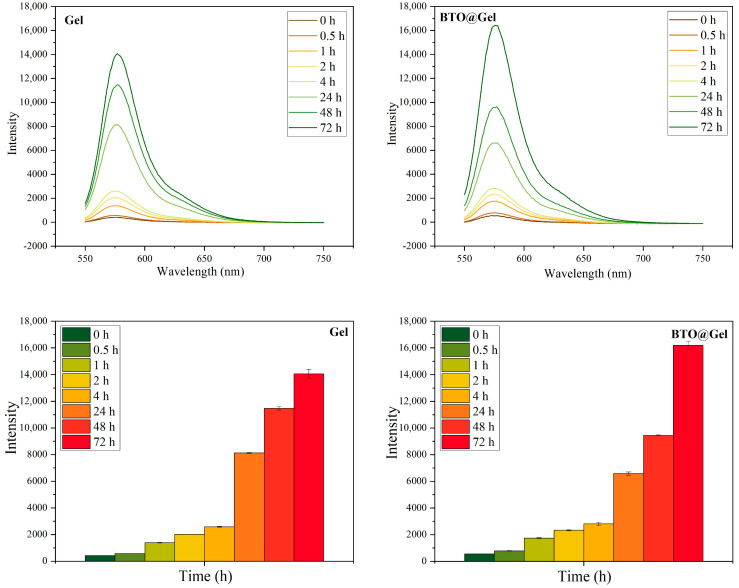
Fluorescence intensity of Gel group and BTO@Gel group at different times. Data are presented as mean ± SD (*n* = 3).

To evaluate the effect of BTO nanoparticles on the controlled release performance of composite hydrogels under ultrasonic stimulation, a series of in vitro experiments was conducted. The underlying mechanism is that, upon ultrasound excitation, BTO—as a piezoelectric material—undergoes lattice deformation, resulting in charge separation and the generation of local electric fields. These fields initiate redox reactions with surrounding water or oxygen molecules, continuously producing ROS [27]. The accumulated ROS then degrade the hydrogel network, inducing structural disintegration and promoting the release of encapsulated substances. Under identical ultrasound conditions, the supernatants from each group were collected and analyzed via fluorescence measurements. As shown in Figure 7, the fluorescence intensity of the experimental group (BTO@Gel) was significantly higher than that of the control group, indicating enhanced hydrogel degradation and a more efficient release of the fluorescent model compound [28]. These results confirm that BTO nanoparticles, when activated by ultrasound, effectively promote ROS generation, thereby accelerating hydrogel disintegration and significantly improving the release efficiency of the composite system.

### 3.5. In Vitro Biological Safety Evaluation of BTO and Its Hydrogel Composites

To assess the in vitro biocompatibility of BTO nanoparticles and their hydrogel composites, the 3T3 mouse embryonic fibroblast cell line was selected as a biosafety evaluation model [29,30]. This adherent cell line is widely used in preliminary cytotoxicity testing due to its stable growth characteristics and high sensitivity to external stimuli. Cell viability was assessed using the Cell Counting Kit-8 (CCK-8) assay, which is based on the WST-8 (2-(2-Methoxy-4-nitrophenyl)-3-(4-nitrophenyl)-5-(2,4-disulfophenyl)-2H-tetrazolium Sodium Salt) colorimetric reaction.

3T3 cells were seeded into 96-well plates and divided into two experimental groups: the BTO group, treated with BTO solutions at varying concentrations (25, 50, 100, 200, 300, 400, and 500 μg/mL), and the BTO@Gel group, treated with extract solutions of gel-coated BTO composite materials at equivalent BTO concentrations matching those of the BTO group. The control group received PBS solution. After 48 h of incubation, CCK-8 reagent was added to each well. Following a 30-min incubation period, the optical density (OD) values were measured, and the relative cell viability was calculated.

**Figure 8:**
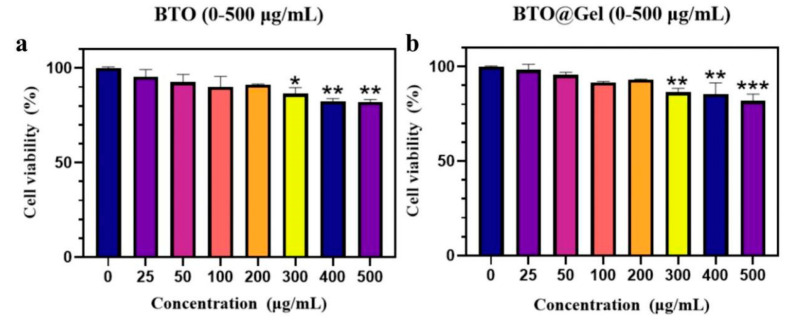
Survival rate of 3T3 cells incubated for 30 min. (**a**) BTO group; (**b**) BTO@Gel group. Data are presented as mean ± SD (n = 3). Statistical significance was determined using one-way ANOVA with Tukey’ s post hoc test. (The asterisks (*) in the text indicate the significance levels of statistical tests, with the specific thresholds as follows: (*) *p* < 0.05, (**) *p* < 0.01, and (***) *p* < 0.001.).

As shown in Figure 8a, even at the highest BTO concentration (500 μg/mL), relative cell viability remained above 80%, indicating low cytotoxicity. Similarly, in the BTO@Gel group (Figure 8b), no significant reduction in viability was observed compared to the control, suggesting that the hydrogel coating did not introduce additional toxicity. Cell viability remained consistently above 80% across all tested concentrations.

In summary, both BTO nanoparticles and their hydrogel composites exhibited good in vitro biocompatibility across the tested concentration range, supporting their potential for further biomedical applications.

## 4. Discussion and Conclusions

In this study, we designed a piezoelectric catalysis-based, ultrasound-responsive hydrogel platform capable of ROS-triggered drug release under acoustic stimulation. This system integrates BTO nanoparticles with a ROS-sensitive hydrogel matrix, enabling controlled release in response to tumor microenvironmental oxidative stress and external ultrasound activation. Our results confirm that BTO nanoparticles generate significant amounts of ^•^OH and ^1^O_2_ under ultrasound, which subsequently induce hydrogel degradation and promote drug release.

Compared to previously reported drug delivery platforms—such as photodynamic therapy (PDT)-activated hydrogels, enzyme-responsive release systems, or passive diffusion-based nanogels—our system offers distinct advantages in spatial precision and remote controllability. Notably, ultrasound has been shown in prior studies to provide greater tissue penetration than light-based modalities, enabling non-invasive activation of embedded therapeutic platforms. While acoustic penetration depth was not directly evaluated in this work, this characteristic makes ultrasound a promising candidate for clinical translation. Furthermore, piezoelectric catalysis eliminates the need for chemical co-factors or specific enzymatic triggers. Recent studies on ROS-based sonodynamic systems support the feasibility of integrating energy conversion with stimulus-responsive drug release, although few have combined these elements within hydrogel matrices as in our approach.

Functionally, we confirmed that BTO-induced ROS generation is predominantly driven by ^•^OH, as evidenced by radical scavenging and fluorescence probe experiments, and that hydrogel disintegration closely correlates with both ROS concentration and ultrasound intensity. In drug release experiments, systems containing BTO exhibited significantly enhanced release under ultrasound compared to control hydrogels, indicating that piezocatalytic activity plays a central role in the release mechanism. Moreover, the biocompatibility of the hydrogel and nanoparticles was evaluated using 3T3 cells, demonstrating acceptable safety at relevant concentrations.

Despite these promising findings, it is important to acknowledge that this study was conducted entirely under in vitro conditions. Due to time and resource constraints, we were unable to evaluate the system under physiological serum conditions, perform high-resolution analyses of nanoparticle–cell interactions, or validate therapeutic efficacy in animal models. These limitations constrain our ability to draw conclusions regarding in vivo specificity, biodistribution, and tumor selectivity. Furthermore, while rhodamine B was employed as a model drug to validate release profiles, future work should incorporate real chemotherapeutic agents and tumor-relevant models to confirm biological activity.

For practical application, the proposed hydrogel system is well suited for intratumoral injection or postoperative cavity administration, particularly in solid tumors requiring localized treatment. The ROS responsiveness and ultrasound-guided activation provide a framework for spatiotemporal control of drug release with minimal systemic exposure. However, to advance toward clinical translation, future research should address key challenges, including in vivo degradation kinetics, immune compatibility, long-term biosafety, and therapeutic efficacy in relevant animal models.

In summary, this study presents a foundational platform integrating piezoelectric catalysis, ROS-responsive materials, and ultrasound activation for controlled drug delivery. While further optimization and validation are required, the system demonstrates considerable promise as a next-generation intelligent biomaterial for tumor therapy.

## Figures and Tables

**Table 1:** Zeta potential and electrophoretic mobility of BTO nanoparticles (*n* = 3).

Sample Name	Zeta Potential (mV)	Mobility (μ/s)/(V/cm)
BTO	−11.18	−0.87
	−12.20	−0.95
	−15.95	−1.25

## Data Availability

The original contributions presented in this study are included in the article; further inquiries can be directed to the corresponding author.

## Abbreviations

The following abbreviations are used in this manuscript:

BTO	Barium Titanate
CCK-8	Cell Counting Kit-8
COMSOL	COMSOL Multiphysics
DOX	Doxorubicin
FFA	Furfuryl Alcohol
HRTEM	High-Resolution Transmission Electron Microscopy
MB	Methylene Blue
NBT	Nitroblue Tetrazolium
NPs	Nanoparticles
OD	Optical Density
PBS	Phosphate-Buffered Saline
pBQ	p-Benzoquinone
ROS	Reactive Oxygen Species
^•^OH	Hydroxyl Radical
^1^O_2_	Singlet Oxygen
O_2_^−^	Superoxide Anion
SOSG	Singlet Oxygen Sensor Green
TA	Terephthalic Acid
TBA	Tert-Butanol
TEM	Transmission Electron Microscopy
XPS	X-ray Photoelectron Spectroscopy
XRD	X-ray Diffraction
